# Molecular biodiversity of cassava begomoviruses in Tanzania: evolution of cassava geminiviruses in Africa and evidence for East Africa being a center of diversity of cassava geminiviruses

**DOI:** 10.1186/1743-422X-2-21

**Published:** 2005-03-22

**Authors:** J Ndunguru, JP Legg, TAS Aveling, G Thompson, CM Fauquet

**Affiliations:** 1 Plant Protection Division, P.O. Box 1484, Mwanza, Tanzania; 2 International Laboratory for Tropical Agricultural Biotechnology, Donald Danforth Plant Science Center, 975 N. Warson Rd., St. Louis, MO 63132 USA; 3 International Institute of Tropical Agriculture-Eastern and Southern Africa Regional Center and Natural Resource Institute, Box 7878, Kampala, Uganda; 4 Department of Microbiology and Plant Pathology, University of Pretoria, Pretoria 0002, South Africa; 5 ARC-Institute for Industrial Crops, Private Bag X82075, Rustenburg 0300, South Africa

**Keywords:** Cassava mosaic disease (CMD), cassava mosaic geminiviruses (CMGs), *African cassava mosaic virus *(ACMV), *East African cassava mosaic virus *(EACMV), *East African cassava mosaic Cameroon virus *(EACMCV), geminivirus recombination, virus evolution.

## Abstract

Cassava is infected by numerous geminiviruses in Africa and India that cause devastating losses to poor farmers. We here describe the molecular diversity of seven representative cassava mosaic geminiviruses (CMGs) infecting cassava from multiple locations in Tanzania. We report for the first time the presence of two isolates in East Africa: (EACMCV-[TZ1] and EACMCV-[TZ7]) of the species *East African cassava mosaic Cameroon virus*, originally described in West Africa. The complete nucleotide sequence of EACMCV-[TZ1] DNA-A and DNA-B components shared a high overall sequence identity to EACMCV-[CM] components (92% and 84%). The EACMCV-[TZ1] and -[TZ7] genomic components have recombinations in the same genome regions reported in EACMCV-[CM], but they also have additional recombinations in both components. Evidence from sequence analysis suggests that the two strains have the same ancient origin and are not recent introductions. EACMCV-[TZ1] occurred widely in the southern part of the country. Four other CMG isolates were identified: two were close to the EACMV-Kenya strain (named EACMV-[KE/TZT] and EACMV-[KE/TZM] with 96% sequence identity); one isolate, TZ10, had 98% homology to EACMV-UG2Svr and was named EACMV-UG2 [TZ10]; and finally one isolate was 95% identical to EACMV-[TZ] and named EACMV-[TZ/YV]. One isolate of *African cassava mosaic virus *with 97% sequence identity with other isolates of ACMV was named ACMV-[TZ]. It represents the first ACMV isolate from Tanzania to be sequenced. The molecular variability of CMGs was also evaluated using partial B component nucleotide sequences of 13 EACMV isolates from Tanzania. Using the sequences of all CMGs currently available, we have shown the presence of a number of putative recombination fragments that are more prominent in all components of EACMV than in ACMV. This new knowledge about the molecular CMG diversity in East Africa, and in Tanzania in particular, has led us to hypothesize about the probable importance of this part of Africa as a source of diversity and evolutionary change both during the early stages of the relationship between CMGs and cassava and in more recent times. The existence of multiple CMG isolates with high DNA genome diversity in Tanzania and the molecular forces behind this diversity pose a threat to cassava production throughout the African continent.

## Background

Geminiviruses are a large family of plant viruses with circular, single-stranded DNA (ssDNA) genomes packaged within geminate particles. The family *Geminiviridae *is divided into four genera (*Mastrevirus*, *Curtovirus*, *Topocuvirus*, and *Begomovirus*) according to their genome organizations and biological properties [1,2]. Members of the genus *Begomovirus *have caused significant yield losses in many crops worldwide [3] and are transmitted by whiteflies (*Bemisia tabaci*) to dicotyledonous plants. The genome of cassava mosaic geminiviruses (CMGs) in the genus *Begomovirus *consists of two DNA molecules, DNA-A and DNA-B, each of about 2.8 kbp [1], which are responsible for different functions in the infection process. DNA-A encodes genes responsible for viral replication [AC1 (*Rep*), and AC3 (*Ren*)], regulation of gene expression [AC2 (*Trap*)] and particle encapsidation [AV1 (*CP*)]. DNA-B encodes for two proteins, BC1 (*MP*) and BV1 (*NSP*) involved in cell-to-cell movement within the plant, host range and symptom modulation [1]. CMGs have been reported from many cassava-growing countries in Africa and the cassava mosaic disease (CMD) induced by them constitutes a formidable threat to cassava production [4].

Representatives of six distinct CMG species have been found to infect cassava in Africa: *African cassava mosaic virus *(ACMV), *East African cassava mosaic virus *(EACMV), *East African cassava mosaic Cameroon virus *(EACMCV), *East African cassava mosaic Malawi virus *(EACMMV), *East African cassava mosaic Zanzibar virus *(EACMZV) and *South African cassava mosaic virus *(SACMV) [5]. Recent studies have uncovered much variation in CMGs including evidence that certain CMGs, when present in mixtures, employ pseudo-recombination or reassortment strategies and recombination at certain hot spots such as the origin of replication [6-10] resulting in the emergence of 'new' viruses with altered virulence. For instance, an ACMV-EACMV recombinant component A, designated EACMV-UG2, and a pseudo-recombinant component B, designated EACMV-UG3 [10], have been implicated in the pandemic of severe CMD currently devastating cassava in much of east and central Africa [4]. In 1997, only ACMV and EACMV were known to occur in Tanzania with the former occurring only in the western part of the country [11]. The discovery of EACMZV on the island of Zanzibar [12] together with the recent spread into Tanzania of the EACMV-UG2 associated pandemic of severe CMD [4,13] has aggravated the CMD situation. Consequently, there is much to be learned about the identity, distribution, molecular variability, and the threat that these emerging geminiviruses pose to cassava production in Tanzania and more generally in Africa.

In 1997, the first recombination between two species of geminiviruses was recorded [7,8]. This mechanism is now known to be widely used by all geminiviruses and is probably the most important molecular mechanism for generating genetic changes that allow novel geminiviruses to exploit new ecological niches [2,14].

This paper describes the results of a molecular study of the sequences of CMGs collected from the major cassava-growing areas of Tanzania in an effort towards identifying, determining molecular variability and mapping the distribution of CMGs. In addition, because East Africa seems to be unusually rich in virus biodiversity and because the most recent cassava pandemic was first reported in East Africa, we investigated the extent of inter-CMG recombinations and examined their role in the evolution of CMGs in Africa.

## Results

### Assessment of CMD symptoms

**Figure 2:**
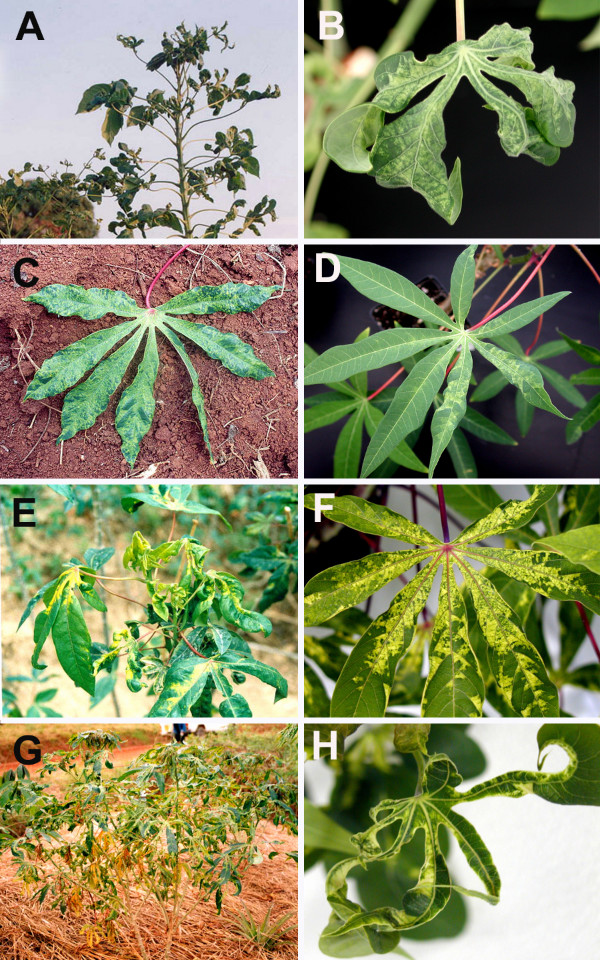
CMD symptoms on naturally infected cassava plants (A, C, E and G) in the field with their corresponding plants raised from field-collected cuttings maintained in the growth chamber (B, D, F and H). Only plants containing single virus infection are shown. Plants A and B contained a single infection of EACMV-[KE/TZM], C and D contained ACMV-[TZ], E and F were infected by EACMCV-[TZ1] and G and H by EACMV-UG2 [TZ10].

Over 80% of the cassava plants in the fields showed severe CMD symptoms with cassava in the Lake Victoria basin expressing the most severe symptoms followed by that from the southern regions. Symptoms of infected cassava samples collected in the field were reproduced in controlled conditions to examine symptom variability. From a total of 35 selected cuttings planted, 25 (71%) were successfully established in the growth chamber. In all cases, regardless of the cultivar, symptoms expressed in the field, whether moderate or severe, were reproduced in the growth chamber and plants did not recover from the disease even 12 months after planting (Fig. 2). Likewise, plants that displayed moderate symptoms in the field showed a similar symptom in the growth chamber as was the case for plants singly-infected with ACMV-[TZ] (Fig. 2).

### Detection of viral genomic components

PCR amplification products (2.7–2.8 kbp) were observed for all the CMG isolates tested using primer UNIF/UNIR (Table 1) designed to amplify near-full-length DNA-A of CMGs. Bands were not observed with the negative control (nucleic acid preparation from healthy cassava plants). Similarly, a specific (2.7 kbp) product was observed when using abutting primers TZ1B-F/R designed from a 560 bp DNA-B fragment initially PCR-amplified using universal primers EAB555/F and EAB555R for general detection of CMGs DNA-B. DNA-B partial fragments (544–560 kbp) were consistently amplified by PCR using primers EAB555-F and EAB555-R (Table 1) for all the CMD-diseased samples previously shown to contain EACMV isolates collected from major cassava-growing areas in Tanzania [13].

**Table 1:** List of the oligonucleotide primers used in this study for amplification of cassava mosaic geminiviruses from Tanzania (^a^nfl = near-full length, ps = partial sequence)

**Primer name**	**Nucleotide sequence (5'→3')**	**Begomovirus isolate**	**DNA component**
UGT-F	TCGTCTAGAACAATACTGATCGGTCTCC	EACMV-KE-[TZT]	DNA-A fl^a^
UGT-R	CGGTCTAGAAGGTGATAGCCGAACCGGGA	EACMV-KE-[TZT]	DNA-A fl
3T-F	ACGTCTAGAACAATACTGATCGGTCTC	EACMV-TZ-[YV]	DNA-A fl
3T-R	GTGCTCTAGAAGGTGATAGCCGAACCGGGA	EACMV-TZ-[YV]	DNA-A fl
TZ1B-F	GCGCGGAATCACTTGTGAAGCAGTCGT	EACMCV-[TZ1]	DNA-B fl
TZ1B-R	GCCGGGATTCGGTGAGTGGTTTACATCAC	EACMCV-[TZ1]	DNA-B fl
EAB555/F	TACATCGGCCTTTGAGTCGCATGG	CMGs	BC1/CR
EAB555/R	CTTATTAACGCCTATATAAACACC	CMGs	BC1/CR
UNI/F	KSGGGTCGACGTCATCAATGACGTTRTAC	CMGs	DNA-A nfl
UNI/R	AARGAATTCATKGGGGCCCARARRGACTGGC	CMGs	DNA-A nfl
AT-F	GTGACGAAGATTGCATTCT	ACMV-[TZ]	DNA-A ps
AT-R	AATAGTATTGTCATAGAAG	ACMV-[TZ]	DNA-A ps
ATZ1-F	TAAGAAGATGGTGGGAATCC	EACMCV-[TZ1]	DNA-A ps
ATZ-R	CGATCAGTATTGTTCTGGAAC	EACMCV-[TZ1]	DNA-A ps
TZ7-F	TGGTGGGAATCCCACCTT	EACMCV-[TZ7]	DNA-A ps
TZ7-R	GTATTGTTATGGAAGGTGATA	EACMCV-[TZ7]	DNA-A ps
TZM-F	TATATGATGATGTTGGTC	EACMV-UG2Svr-[TZ10]	DNA-A ps
TZ10-R	TAGAAGGTGATAGCCGTA	EACMV-UG2Svr-[TZ10]	DNA-A ps
TZM-F	TATATGATGATGTTGGTC	EACMV-KE-[TZM]	DNA-A ps
TZM-R	TAGAAGGTGATAGCCGAAC	EACMV-KE-TZM]	DNA-A ps

### Complete nucleotide sequence characteristics of CMGs from Tanzania

**Figure 3:**
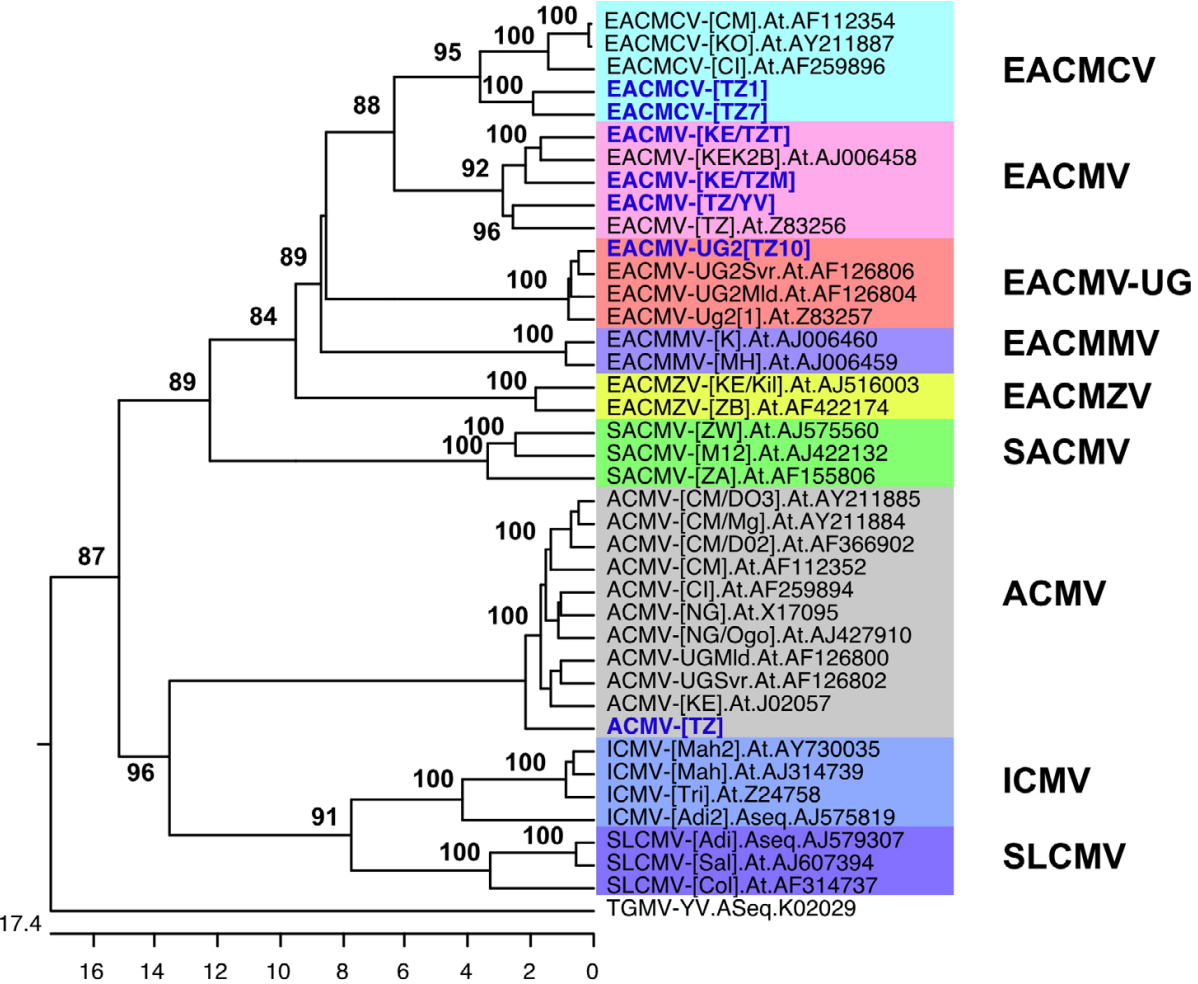
Phylogenetic tree (1000 boot strap replications) showing the DNA-A complete nucleotide sequence relationships between the seven Tanzanian cassava mosaic geminivirus isolates (in blue) and other cassava mosaic geminiviruses. *Tomato golden mosaic virus *(TGMV-YV) (K02029) was used as the out group. Abbreviations and accession numbers are: ACMV-[CI], *African cassava mosaic virus*-[Côte d'Ivoire] (AF259894); ACMV-[NG/Ogo], *African cassava mosaic virus*-[Nigeria-Ogo] (AJ427910); ACMV-[CM/D02], *African cassava mosaic virus*-[Cameroon D02] (AF366902); ACMV-[CM/D03], *African cassava mosaic virus*-[Cameroon D03] (AY211885); ACMV-[CM/Mg], *African cassava mosaic virus*-[Cameroon Mg] (AY211884); ACMV-[CM], *African cassava mosaic virus*-[Cameroon] (AF112352); ACMV-[KE], *African cassava mosaic virus*-[Kenya] (J02057); ACMV-[NG], *African cassava mosaic virus*-[Nigeria] (X17095); ACMV-UGMld, *African cassava mosaic virus*-Uganda mild (AF126800); ACMV-UGSvr, *African cassava mosaic virus*-Uganda severe (AF126802); EACMCV-[CM/KO], *East African cassava mosaic Cameroon virus*-[Cameroon KO] (AY211887); EACMCV-[CM], *East African cassava mosaic Cameroon virus*-[Cameroon] (AF112354); EACMCV-[CI], *East African cassava mosaic Cameroon virus*-[Côte d'Ivoire] (AF259896); EACMMV-[K], East *African cassava mosaic Malawi virus*-[K] (AJ006460); EACMMV-[MH], *East African cassava mosaic Malawi virus*-[MH] (AJ006459); EACMV-[KE/k2B], *East African cassava mosaic virus *[Kenya-K2B] (AJ006458); EACMV-[TZ], *East African cassava mosaic virus*-[Tanzania] (Z53256); EACMV-UG2[2], *East African cassava mosaic virus*-Uganda2[2] (Z83257); EACMV-UG2Mld, *East African cassava mosaic virus*-Uganda2 mild (AF126804); EACMV-UG2Svr, *East African cassava mosaic virus*-Uganda2 severe (AF126806); EACMZV-[KE/Kil], *East African cassava mosaic Zanzibar virus*-[Kenya -Kil] (AJ516003); EACMZV-[ZB], *East African cassava mosaic Zanzibar Virus *– [Zanzibar] (AF422174); ICMV-[Adi2], *Indian cassava mosaic virus *– [Adivaram 2] (AJ575819); ICMV-[Mah], *Indian cassava mosaic virus *– [Maharashstra] (AJ314739); ICMV-[Mah2], *Indian cassava mosaic virus *– [Maharashstra 2] (AY730035); ICMV-[Tri], *Indian cassava mosaic virus *– [Trivandrum] (Z24758); SACMV-[M12], *South African cassava mosaic virus*-[Madagascar M12] (AJ422132); SACMV-[ZA], *South African cassava mosaic virus *– [South Africa] (AF155806); SACMV-[ZW], *South African cassava mosaic virus *– [Zimbabwe] (AJ575560); SLCMV-[Adi], *Sri-Lankan cassava mosaic virus*-[Adivaram] (AJ579307); SLCMV-[Col], Sri-Lankan cassava mosaic virus-[Colombo] (AF314737); SLCMV-[Sal], *Sri-Lankan cassava mosaic virus*-[Salem] (AJ607394).

The complete DNA-A sequences of seven representative CMGs from the major cassava-growing areas were determined from the representative isolates selected and grown in the growth chambers. An ACMV isolate from Tanzania (ACMV-[TZ]) was shown to be most closely related to ACMV-UGMld from Uganda with a sequence identity of 97%. Its DNA-A nucleotide (nt) sequence was established to be 2779 nts in length. It has a high overall sequence identity (> 90%) with all other published sequences of ACMV isolates (Table 2) with which it clusters in the phylogenetic tree presented in Figure 3. The DNA-A sequence organization was typical of a begomovirus, with two open reading frames (ORFs) (AV2 and AV1) in the virion-sense DNA, and four ORFs (AC1 to AC4) in the complementary sense, separated by an intergenic region (IR). Complete nt sequences of the DNA-A genomes of the different Tanzanian EACMV and ACMV isolates were compared with published sequences (Table 2).

**Table 2:** Nucleotide sequence identities (percentages) of the DNA-A full-length of cassava mosaic geminiviruses from Tanzania and other geminiviruses from Africa and the Indian sub-continent. Values above 89% are in bold and names of isolates from Tanzania are in bold.

Virus Isolate	**ACMV-[TZ]**	**EACMCV-[TZ1]**	**EACMCV-[TZ7]**	**EACMV-[KE/TZT]**	**EACMV-[KE/TZM]**	**EACMV-[TZ/YV]**	**EACMV-UG2 [TZ10]**
ACMV-[CM]	**95**	68	68	70	70	69	73
ACMV-[CM/DO2]	**95**	68	68	70	70	69	73
ACMV-[IC]	**96**	68	68	70	71	70	73
ACMV-[KE]	**96**	68	68	70	70	70	73
ACMV-[NG]	**95**	68	68	70	70	70	73
ACMV-[NG/Ogo]	**96**	68	68	70	70	70	73
ACMV-UGMld	**97**	68	68	70	71	70	73
ACMV-UGSVr	**96**	68	68	70	71	70	74
**ACMV-[TZ]**	-	68	68	70	70	70	73

EACMCV-[CM]	67	**90**	**89**	87	87	85	84
EACMCV-[CI]	67	**90**	**90**	88	87	86	85
**EACMCV-[TZ1]**	68	-	**96**	88	88	87	85
**EACMCV-[TZ7]**	68	**96**	-	88	88	87	85

EACMMV-[K]	71	**81**	**81**	**87**	**88**	**86**	**87**
EACMMV-[MH]	71	**81**	**81**	**87**	**88**	**86**	**88**

EACMV-[KE/K2B]	70	88	88	**97**	**96**	**94**	**92**
EACMV-[TZ]	69	88	88	**94**	**94**	**95**	**91**
**EACMV-[KE/TZT]**	70	88	88	**-**	**95**	**93**	**92**
**EACMV-[KE/TZM]**	70	88	88	**96**	-	**94**	**92**
**EACMV-[TZ/YV]**	70	87	87	**94**	**93**	**-**	**90**
EACMV-UG2	73	85	85	**92**	**92**	**92**	**98**
EACMV-UG2Mld	73	86	86	**93**	**92**	**92**	**99**
EACMV-UG2Svr	73	86	86	**93**	**92**	**92**	**99**
**EACMV-UG2 [TZ10]**	73	85	85	**92**	**92**	**91**	**-**

EACMZV-[ZB]	72	80	80	86	86	86	83
EACMZV-[KE/Kil]	72	79	79	86	86	85	83

SACMV-[ZA]	74	73	73	80	80	79	80
SACMV-[ZW]	74	73	73	80	80	80	80
SACMV-[M12]	74	73	73	80	80	80	80

SLCMV-[Col]	73	67	67	67	67	67	67

TGMV-[Com]	58	59	59	59	59	59	59

**Figure 4:**
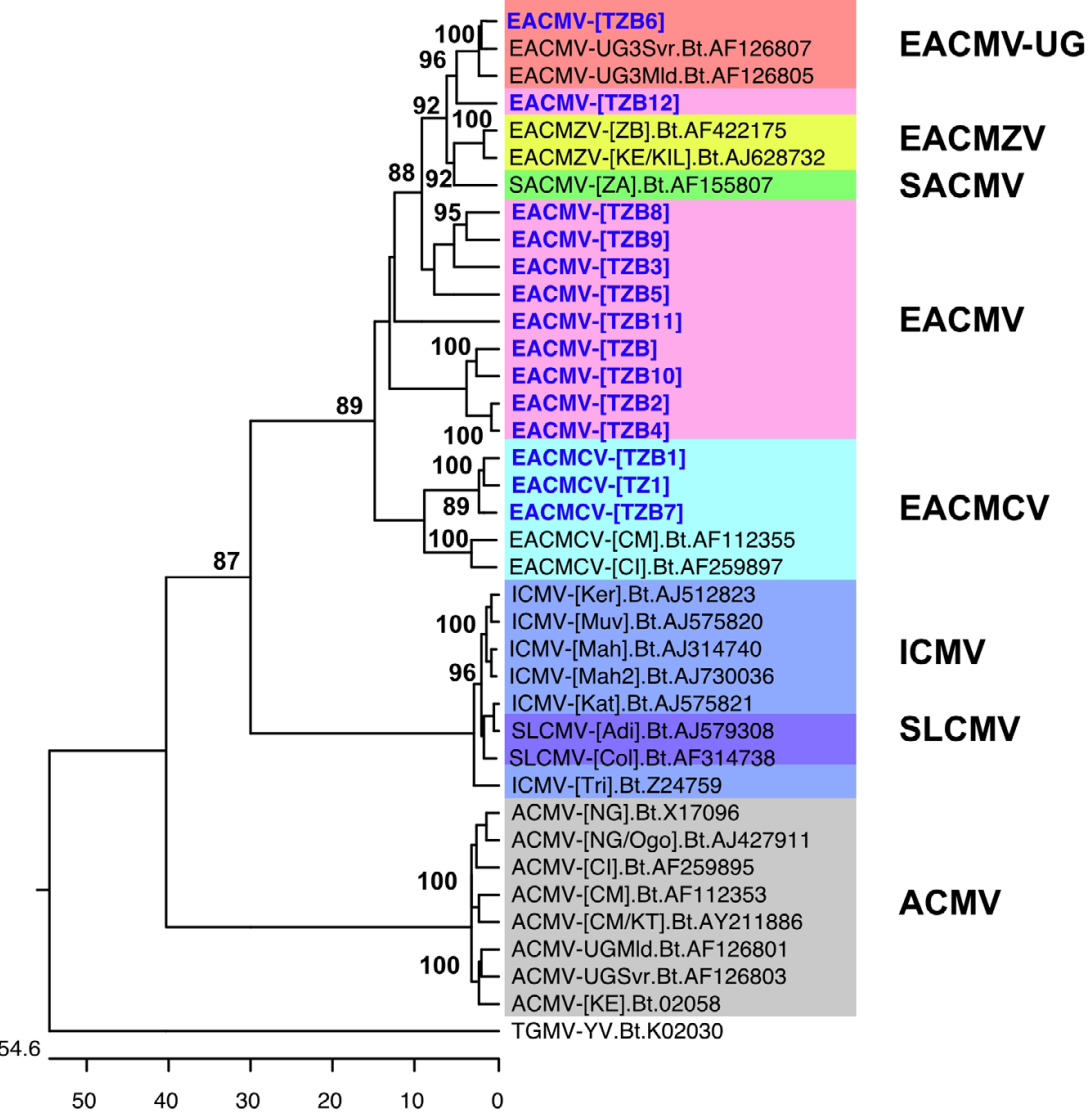
Phylogenetic tree (1000 bootstrap replications) obtained from comparison of the complete nucleotide sequence of EACMCV-[TZ1] DNA-B, partial B component sequences from Tanzania (TZBx) and available cassava mosaic geminivirus DNA-B component sequences. *Tomato golden mosaic virus *(TGMV-YV) (K02030) was used as the out-group. Abbreviations and accession numbers are: ACMV-[CI], *African cassava mosaic virus*-[Côte d'Ivoire] (AF259895); ACMV-[NG/Ogo], *African cassava mosaic virus*-[Nigeria-Ogo] (AJ427911); ACMV-[CM/KT], *African cassava mosaic virus*-[Cameroon KT] (AY211886); ACMV-[CM], *African cassava mosaic virus*-[Cameroon] (AF112353); ACMV-[KE], *African cassava mosaic virus*-[Kenya] (J02058); ACMV-[NG], *African cassava mosaic virus*-[Nigeria] (X17096); ACMV-UGMld, *African cassava mosaic virus*-Uganda mild (AF126801); ACMV-UGSvr, *African cassava mosaic virus*-Uganda severe (AF126803); EACMCV-[CM], *East African cassava mosaic Cameroon virus*-[Cameroon] (AF112355); EACMCV-[CI], *East African cassava mosaic Cameroon virus*-[Côte d'Ivoire] (AF259897); EACMV-UG3Mld, *East African cassava mosaic virus*-Uganda3 mild (AF126805); EACMV-UG3Svr, *East African cassava mosaic virus*-Uganda3 severe (AF126807); EACMZV-[KE/Kil], *East African cassava mosaic Zanzibar virus*-[Kenya -Kil] (AJ628732); EACMZV-[ZB], *East African cassava mosaic Zanzibar Virus *– [Zanzibar] (AF422175); ICMV-[Kat], *Indian cassava mosaic virus *– [Kattukuda] (AJ575821); ICMV-[Ker], *Indian cassava mosaic virus *– [Kerala] (AJ575823); ICMV-[Mah], *Indian cassava mosaic virus *– [Maharashstra] (AJ314740); ICMV-[Mah2], *Indian cassava mosaic virus *– [Maharashstra 2] (AY730036); ICMV-[Tri], *Indian cassava mosaic virus *– [Trivandrum] (Z24759); SACMV-[ZA], *South African cassava mosaic virus *– [South Africa] (AF155807); SLCMV-[Adi], *Sri-Lankan cassava mosaic virus*-[Adivaram] (AJ579308); SLCMV-[Col], *Sri-Lankan cassava mosaic virus*-[Colombo] (AF314738).

Two isolates, TZ1 and TZ7, with 2798 and 2799 nts respectively, collected from Mbinga district in southwestern Tanzania, were most closely related to isolates of the species *East African cassava mosaic Cameroon virus *from Cameroon and Ivory Coast, West Africa, (EACMCV-[CM], -[CI]), with 89–90% nt sequence identity. They are clearly isolates of EACMCV and we have named them EACMCV-[TZ1] and EACMCV-[TZ7] to indicate that they were from Tanzania and to distinguish them from the original EACMCV-[CM] isolate from Cameroon. The two isolates were also virtually identical to one another having high overall DNA sequence conservation (93% nt sequence identity). Phylogenetic analysis of the DNA-A nt sequences grouped EACMCV-[TZ1] and EACMCV-[TZ7] in the same cluster with EACMCV-[CM] and EACMCV-[CI] (Fig. 3). The complete nt sequence of the EACMCV-[TZ1] DNA-B component was determined to be 2726 nts long and had the highest sequence identity (85%) with EACMCV-[CM] DNA-B with which it is grouped in the phylogenetic tree (Fig. 4). It had less than 72% homology with DNA-Bs of other EACMV isolates from East Africa.

The complete DNA-A genome of CMG isolates from Yombo Vituka (YV) and Tanga (TZT) in the coastal area of Tanzania were determined to be 2800 and 2801 nts long respectively. Isolate YV showed high (95%) overall nt sequence identity with previously characterized EACMV-[TZ] and is therefore named EACMV-[TZ/YV] in the Dar-es-Salaam region. It also had high overall sequence identity (87–96%) with other Tanzanian EACMV isolates characterized in this study (Table 2). Phylogenetic analysis of the complete nt sequence of EACMV-[TZ/YV] grouped it with its closest relative, EACMV-TZ (Fig. 3). CMG isolate TZT had high sequence identity (96.5%) with EACMV-[KE/K2B] from Kenya and is named EACMV-[KE/TZT]. Similarly, another CMG isolate (TZM) from the Mara region in the Lake Victoria zone was found to have high overall sequence identity (96%) with EACMV-[KE/K2B] and we have named it EACMV-[KE/TZM]. This isolate, 2805 nts in length, together with EACMV-[KE/TZT], clustered with EACMV-[KE/K2B] in the phylogenetic tree (Fig. 3). Another isolate from Kagera region in northwestern Tanzania (TZ10) showed very high overall DNA-A nt sequence identity (98.8%) with the published sequence of EACMV-UG2Svr. Its complete DNA-A nt sequence was 2804 nts long and it was named EACMV-UG2 [TZ10].

### Determination of genetic diversity of EACMV DNA-B using partial sequences

The diversity of different CMG isolates was analyzed using a partial DNA-B genomic region spanning the N-terminal region of BC1 to the intergenic region (IR). Identities of these sequences with those of the corresponding DNA-B genomic regions of other CMGs in GenBank were determined. Generally, the EACMV isolates showed little genetic divergence amongst one another and isolates collected from the same area displayed high nt sequence identity. Isolates TZB1 and TZB7 from the southern part of Tanzania shared the highest (98%) nt sequence identity followed by TZB3 and TZB8 (94%) as well as TZB and TZB10, all from the east coast area. TZB2 was most closely related to and shared 91% sequence identity with TZB4, both collected from the coastal area. None of the isolates from the south or coastal areas shared >85% nt sequence identity with those from the Lake Victoria basin (TZB9 and TZB12).

The phylogenetic tree generated from a multiple alignment of 13 EACMV isolates with selected bipartite begomovirus sequences and EACMCV-[TZ1] B component is shown in Figure 4. All 13 Tanzanian isolates studied clustered with the reference EACMVs, with TZB6 being most closely related to Ugandan isolates (EACMV-UG3Svr, EACMV-UG3Mld and EACMV-UG1) (Fig. 4) sharing 97% nt sequence identity. Four isolates (TZB3, TZB5, TZB8 and TZB9) formed a closely related group, with TZB8 and TZB9 being the most closely related. Isolates TZMB, TZB5 and TZB11 each grouped separately. None of the EACMV isolates grouped with ICMV and SLCMV from the Indian subcontinent (Fig. 4).

### Capsid protein (CP) gene sequence analysis and comparison with selected viruses

The CP gene sequences of the seven CMGs identified in our study were compared to published sequences (Table 3). ACMV-[TZ] shared the highest nt sequence identity (97.4%) with ACMV-UGMld from Uganda followed by ACMV-[CM], an isolate from Cameroon. The lowest sequence identity (63.2%) was recorded with TGMV-YV (Table 3), an American begomovirus. Both EACMCV-[TZ1] and EACMCV-[TZ7] were more than 92% identical to EACMCV-[CM], but they also had very high nt sequence identity (95%) with EACMZV from Zanzibar and EACMV-[KE/K2B] (Table 3) and 96% between each other. Interestingly, EACMV-[KE/TZT] and EACMV-[KE/TZM] collectively shared high (97%) identity with EACMZV followed by EACMV-[KE/K2B](96–97%) and up to 96% between each other. Furthermore the EACMV-[TZ/YV] CP gene sequence showed very high identity with EACMV-[TZ] (96%) and EACMZV (96%) followed by EACMV-[KE/K2B](95%) (Table 3). The EACMV-UG2 [TZ10] sequence shared a very high nt sequence identity (99%) with EACMV-UG2Svr from Uganda and high identity (98–99%) with other Ugandan isolates of EACMV. As expected, EACMV-UG2 [TZ10] shared 90% sequence homology with ACMV (Table 3), suggesting it contained the recombination at the CP gene level previously reported [7,8] for EACMV-UG2.

**Table 3:** CP gene nucleotide sequence identity (%) of cassava mosaic geminiviruses from Tanzania and other published CMG CP sequences. Values above 89% are in bold and names of isolates from Tanzania are in blue.

Virus Isolate	**ACMV-[TZ]**	**EACMCV-[TZ1]**	**EACMCV-[TZ7]**	**EACMV-[KE/TZT]**	**EACM-[KE/TZM]**	**EACMV-[TZ/YV]**	**EACMV-UG2 [TZ10]**
ACMV-[CM]	**97**	77	77	78	79	77	**90**
ACMV-[CI]	**96**	77	78	78	79	77	**90**
ACMV-[KE]	**97**	76	76	77	78	76	**90**
ACMV-[NG]	**96**	77	77	78	78	77	**90**
ACMV-UGMld	**97**	76	77	78	78	76	**90**
**ACMV-[TZ]**	-	77	77	78	78	77	**89**

EACMCV-[CM]	77	**94**	**94**	**95**	**96**	**93**	84
**EACMCV-[TZ1]**	77	-	**97**	**95**	**96**	**94**	84
**EACMCV-[TZ7]**	77	**97**	-	**95**	**97**	**95**	84

EACMMV-[K]	77	80	80	80	80	80	79
EACMMV-[MH]	77	79	80	80	80	80	79

EACMV-[KE/K2B]	77	**95**	**96**	**96**	**97**	**96**	84
EACMV-TZ	77	**95**	**95**	**96**	**97**	**96**	85
**EACMV-[KE/TZT]**	78	**95**	**95**	-	**97**	**95**	85
**EACMV-[KE/TZM]**	78	**96**	**97**	**97**	**-**	**97**	84
**EACMV-[TZ/YV]**	77	**94**	**95**	**95**	**96**	**-**	84
EACMV-UG2	**90**	84	84	85	85	84	**99**
EACMV-UG2Mld	**89**	84	84	85	85	84	**98**
EACMV-UG2Svr	**90**	84	84	85	85	84	**99**
**EACMV-UG2 [TZ10]**	**89**	84	84	85	84	84	**-**

EACMZV-[ZB]	78	**96**	**96**	**97**	**97**	**96**	85

SACMV-[ZA]	77	78	79	80	79	79	73

ICMV-[Tri]	74	73	73	74	74	73	64

TGMV-[Com]	63	65	65	64	64	65	78

**Figure 5:**
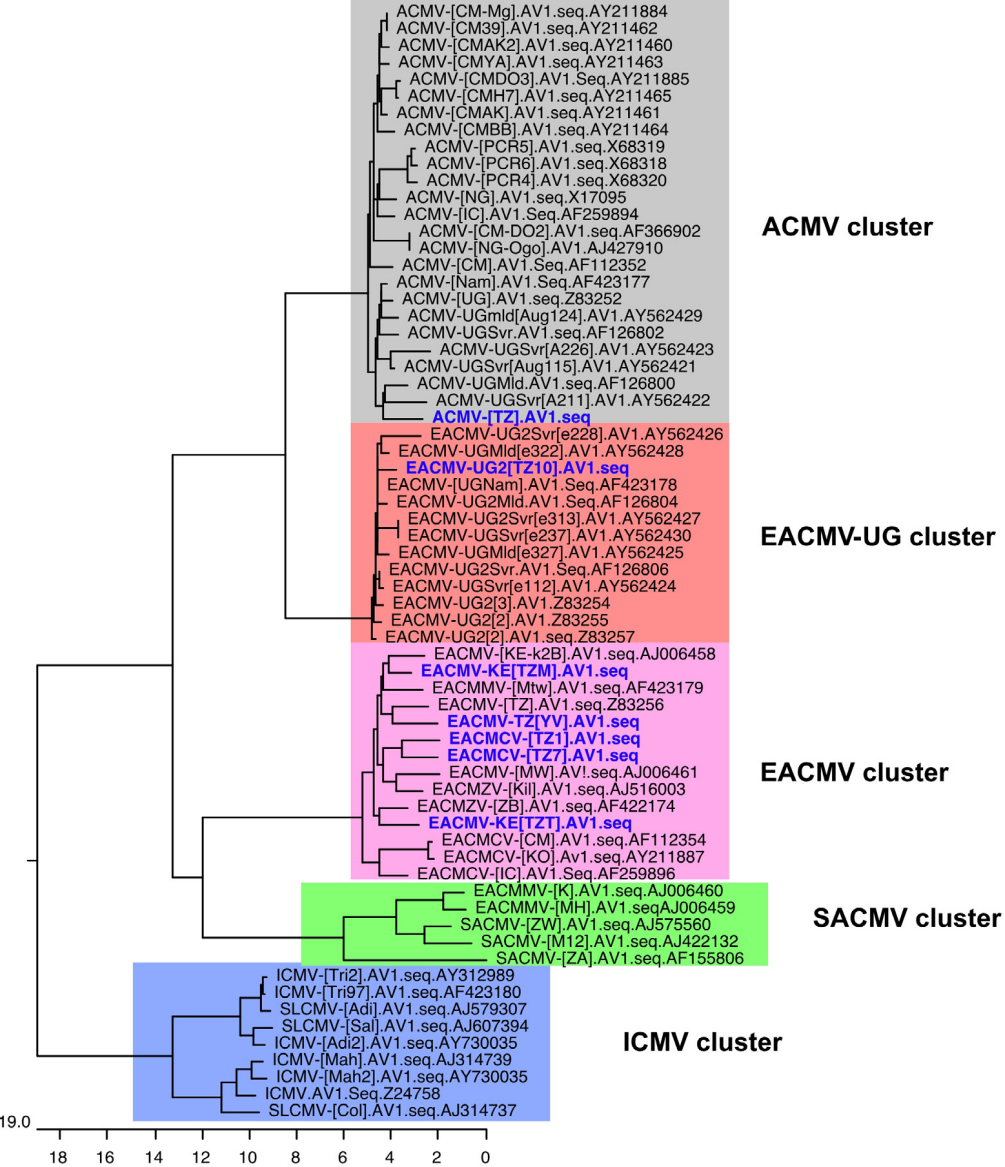
Phylogenetic tree of the coat protein gene (CP) nucleotide sequences of the cassava mosaic geminivirus isolates from Tanzania and other cassava begomoviruses (1000 bootstrap replications). Sequence of tomato golden mosaic virus (TGMV-YV) was used as the out-group. Abbreviations and accession numbers can be found in Figure 3.

A phylogenetic analysis of the CP of Tanzanian CMGs yielded a tree (Fig. 5) that was in agreement with the relationship predicted by pairwise sequence comparison (Table 4). ACMV-[TZ] clustered with other ACMV isolates while EACMV-UG2 [TZ10] grouped with Ugandan isolates of EACMV. EACMCV-[TZ1], EACMCV-[TZ7], EACMV-[TZ/YV], and the two viruses, EACMV-[KE/TZT] and EACMV-[KE/TZM] clustered with other EACMV isolates from either Cameroon or Kenya. No CMG isolate identified in this study clustered with EACMMV from Malawi, SACMV from South Africa, ICMV, or SLCMV from the Indian sub-continent when their CP gene nucleotide sequences were compared (Fig. 5).

**Table 4:** Percent similarity (in the upper triangle) in the nucleotide sequence of the common region of East and West African isolates of EACMCV. Values of 89% and above are in bold.

Virus isolate	**EACMCV-[TZ1] CRA**	**EACMCV-[TZ7] CRA**	**EACMCV-[TZ1] CRB**	**EACMCV-[CM] CRA**	**EACMCV-[CM] CRB**	**EACMCV-[CI] CRA**	**EACMCV-[IC] CRB**
**EACMCV-[TZ1] CRA**	***	80	80	**89**	76	82	76
**EACMCV-[TZ7] CRA**		***	86	88	74	82	73
**EACMCV-[TZ1] CRB**			***	**91**	80	82	78
**EACMCV-[CM] CRA**				***	86	**91**	83
**EACMCV-[CM] CRB**					***	78	**97**
**EACMCV-[CI] CRA**						***	77
**EACMCV-[CI] CRB**							***

### The common regions (CRs) of the Tanzanian CMGs

**Figure 6:**
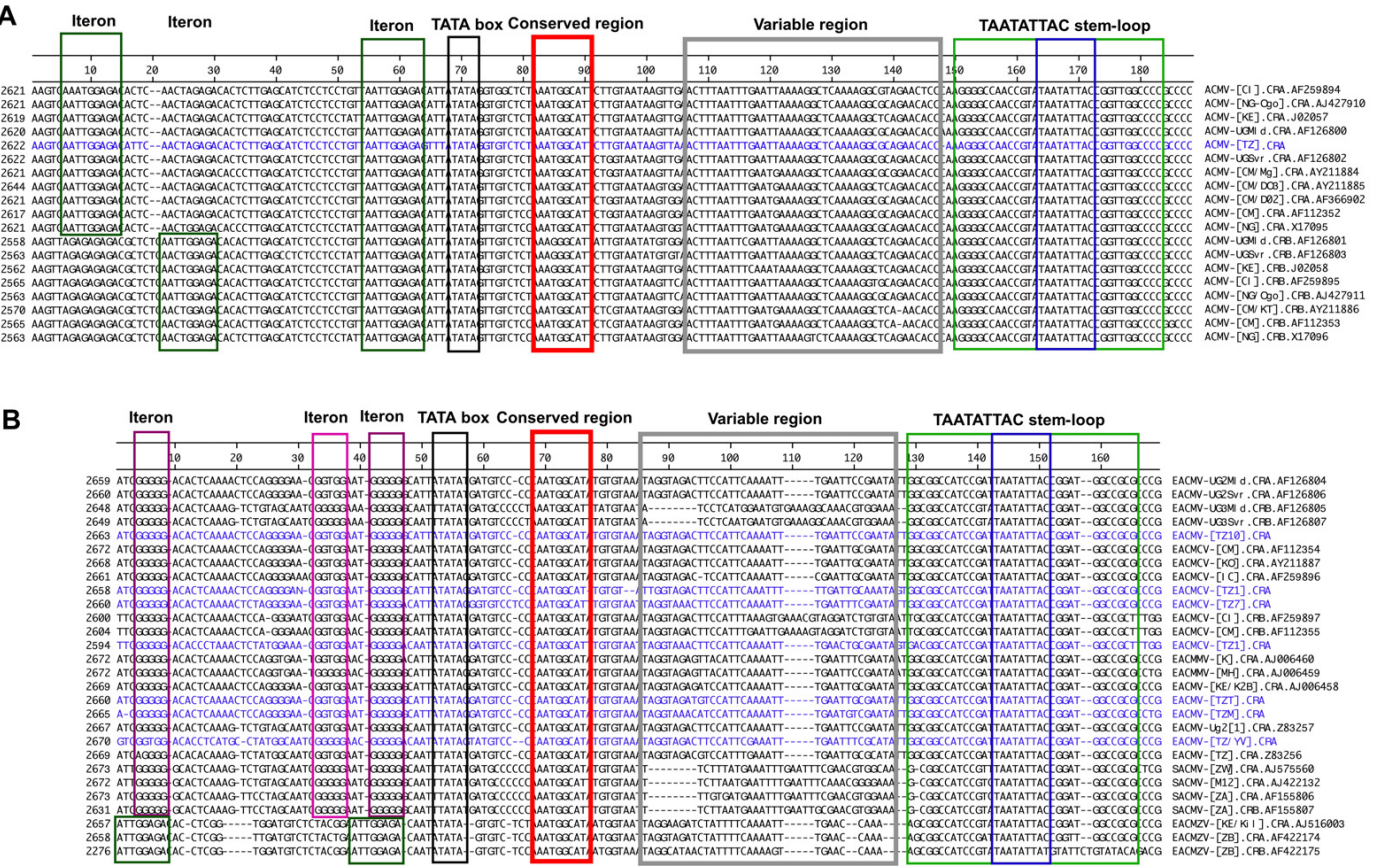
Alignment of common region (CR) nucleotide sequences of the DNA-A (CRA) and DNA-B (CRB) of ACMV (A) and EACMV (B) isolates from Tanzania with the related isolates of ACMV and EACMV from the database sequences. The TATA box for AC1 is boxed in black. The putative Rep binding iterative sequences (iterons) are boxed in green and purple. The conserved nonanucleotide sequences TAATATTAC together with its stem loop are boxed in blue and green respectively. The conserved sequence 3'-end of the TATA box is boxed in red and the so-called "variable region" is boxed in grey. Virus sequences from Tanzania are written in blue. The accession numbers of the sequences from GenBank are indicated on the right of the virus abbreviation names and the significance of these abbreviations can be found in the legend of Figures 3 and 4.

The conserved nonanucleotide in the hairpin-loop, TAATATTAC, that is characteristic of the members of the family *Geminiviridae *and the AC1 TATA box, were identified in the CR sequences of all the Tanzanian CMGs (Fig. 6a,6b). The CR of ACMV-[TZ] was 170 nts long while those for EACMV were between 152 and 157 nts in length. When the CR sequence of ACMV-[TZ] was compared and aligned to the published CR sequences of other cassava-infecting ACMV isolates from Africa (Fig. 6a), it was apparent that ACMV-[TZ] was virtually identical to all ACMV isolates. The repeated motif upstream the TATA box for all the published ACMV isolates was AATTGGAGA (Fig. 6a). The motif for ACMV-[TZ], AATTGGAGA, was identical. Figure 6b presents the alignment of the CRs of the Tanzanian EACMVs with sequences of all published EACMVs. It was found that all the isolates contained the various features characteristic of begomoviruses. The putative Rep-binding sequences (iterons) were GGTGGAATGGGGG for all the Tanzanian isolates except EACMV-[TZ/YV] that had different iterons (GGGGGAACGGGGG) and a total of 23 mismatches in the entire CR. It is worth noting that although the genomes of the two isolates of EACMZV are EACMV-based, their CRs are more similar to ACMV than to EACMV and the iteron is AATTGGAGA.

The comparisons of the nt sequences of the CRs of Tanzanian CMGs with other CMGs revealed high sequence identity (> 90%) of ACMV-[TZ] to published sequences of other ACMV isolates and low identity (61–62%) to EACMV species. Similarly, all the Tanzanian EACMV isolates were related with sequence identities of 83–97% between CRs of the DNA-A and DNA-B. The CR of EACMV-[TZ/YV] showed a relatively low sequence identity to other isolates. EACMCV-[TZ1] (DNA-A and -B) and the EACMCV-[TZ7] showed high nt sequence identity to EACMCV (Table 4).

### Geographical distribution of the CMGs in Tanzania

**Figure 7:**
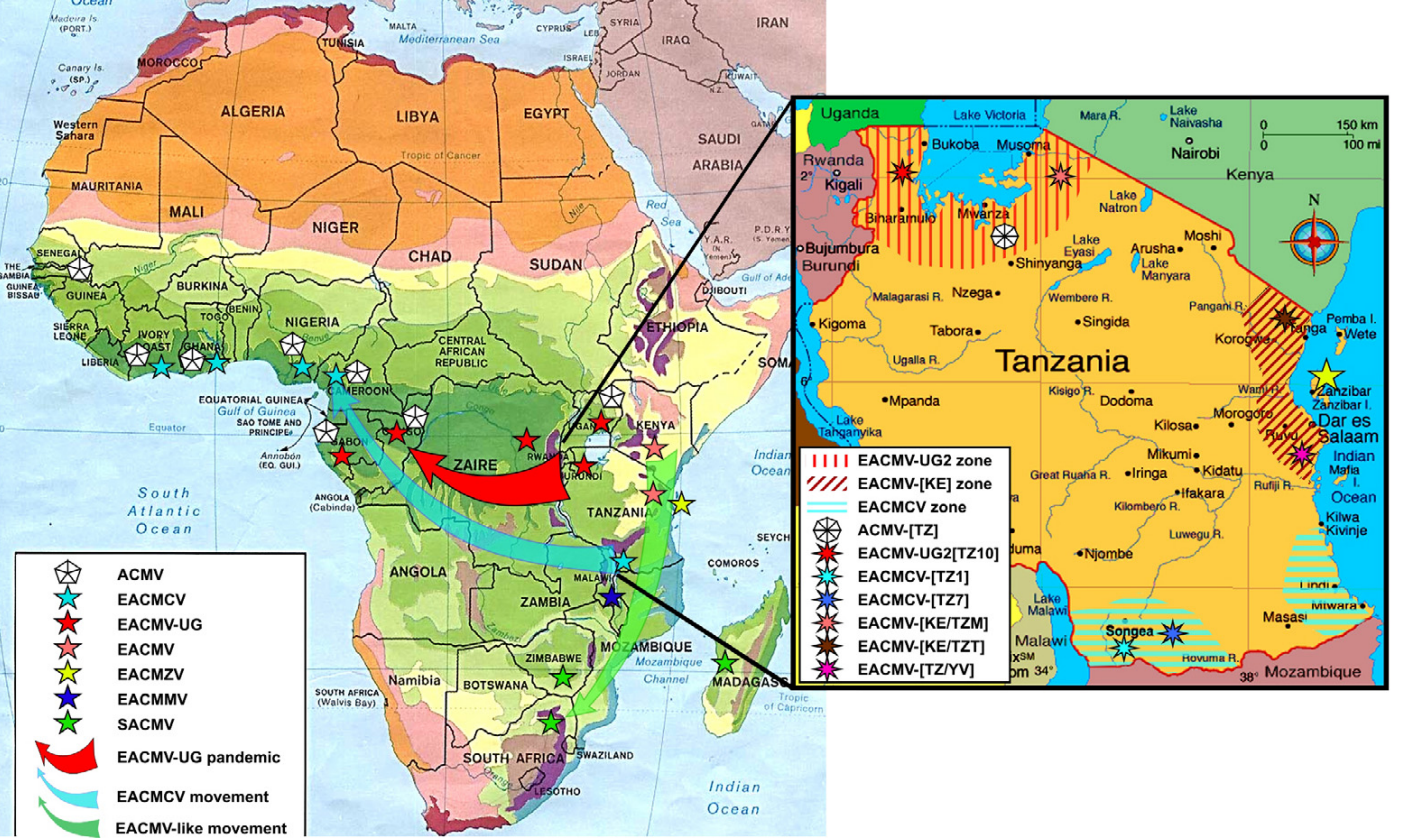
Map of the location of the different types of viruses present in Africa and inlay map of Tanzania showing the location of the completely sequenced CMG clones in that study as well as the localization of the distribution of viruses similar to these clones by RFLP mapping [13]. On the African map the symbols represent an approximate positioning of the viruses for which we have complete sequence information and not those for which we have either partial sequence information or serological data only. The significance of the different stars and shaded areas and arrows is indicated in the legend boxes in the figure. The solid red arrow represents the current direction of spread of the CMD pandemic, while the faded green and blue arrows represent possible "routes" of evolution of EACMV-like viruses and EACMCV in the past.

The representative isolates sequenced here have been chosen because they represent a range of different RFLP patterns found during a large set of 485 samples collected throughout Tanzania [13]. However, the selection of isolates to sequence was based on the differences in RFLP patterns and not on their frequency of appearance in the country. Figure 7 shows the different locations of these samples represented by the isolates sequenced here. The EACMCV-[TZ1] was the most widespread, found in 50 samples located mainly in the southern part of Tanzania in the Mbinga District of Ruvuma Region. EACMCV-[TZ7], the close relative of EACMCV-[TZ1], was found only in one sample in the same district of Mbinga. EACMV-[KE/TZT] was found only in the coastal areas, in ten samples, mainly in Tanga and Pwani regions. EACMV-[KE/TZM] was found in ten samples, only in the Mara Region of the Lake Victoria Basin and to a very limited extent on the island of Ukerewe in Lake Victoria. The rest of the CMGs, EACMV-UG2 [TZ10], ACMV-[TZ] as well as EACMV-[TZ/YV], had a limited geographical distribution (Fig. 7).

### Comparisons of the East African and West African isolates of EACMCV

#### i) Comparisons of the A components of EACMCV-[TZ]

**Figure 8:**
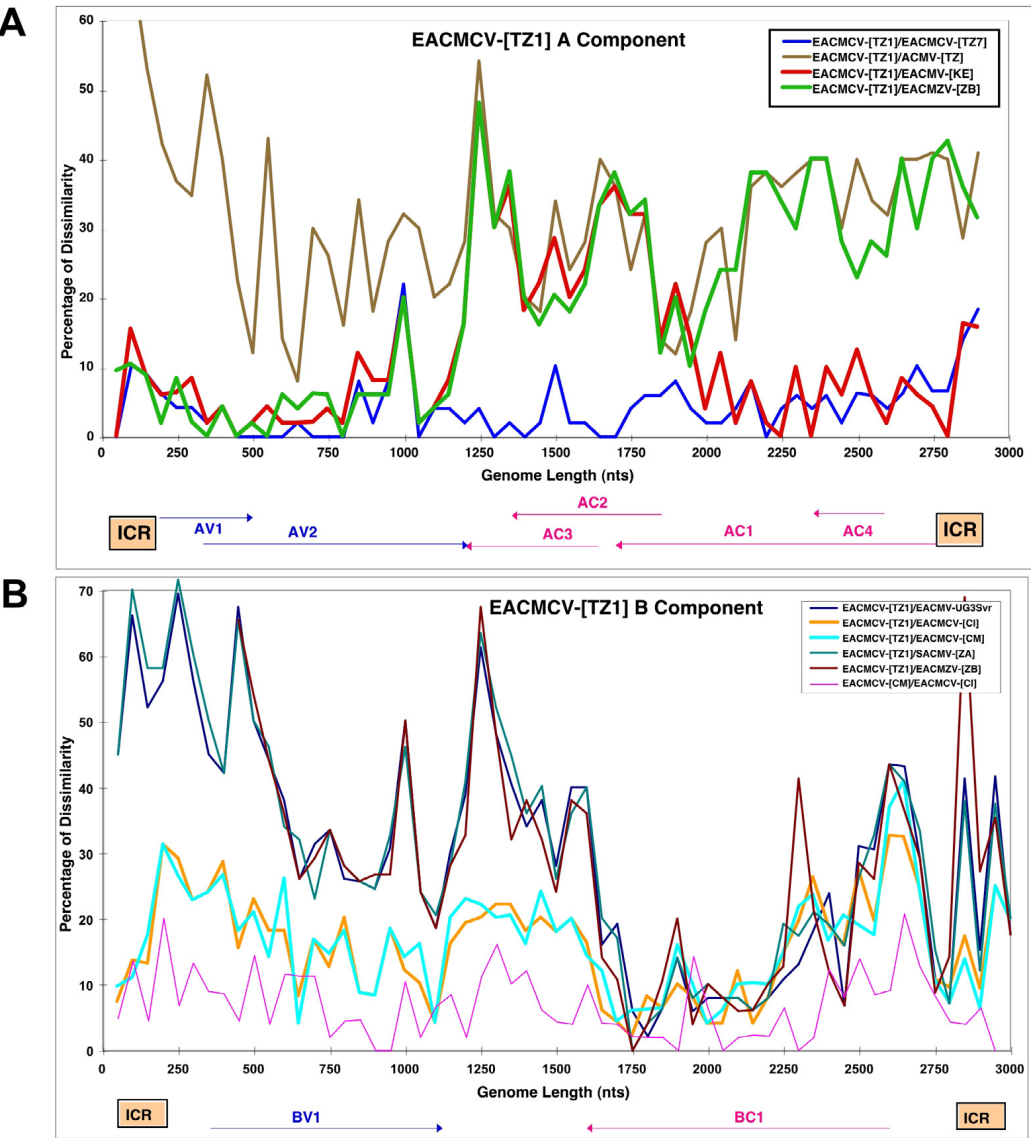
Pairwise sequence comparisons of EACMCV-[TZ1, TZ7] DNA-A (A) and DNA-B (B). Each curve represents a sequence comparison along the linearized virus genomes of a chosen pair of viruses. The correspondance for each colored curve is given in the figure. The dissimilarity index (Y axis) is the percentage of dissimilarity over a window of 50 nucleotides. The curves under 10% represent a pair of isolates of the same species and curves above 10% represent a pair of isolates belonging to different species. A switch between the two types of curves represents a putative recombination between the two viruses or their ancestors. The linearized genome organization of each component is depicted at the bottom.

The *East African cassava mosaic Cameroon virus *isolates from Tanzania (EACMCV-[TZ1, TZ7]) are very typical isolates of the species *East African cassava mosaic Cameroon virus*. The A component was 89 to 90% identical to the isolates from Cameroon and Ivory Coast and the 300 nts that differ are scattered all along the genome. In addition, the A components from East Africa showed the typical recombination already noted in the West African isolates, *i.e. *a fragment of about 800 nts not of EACMV origin, covering AC2-AC3 and the C-terminus of AC1 (Fig. 8A).

#### ii) Comparisons of the B components of EACMCV

The EACMCV West African isolates had only a stretch of 800 nts in the BC1 region in common with EACMV isolates from Uganda, the only B component available for EACMV: the rest of the sequence was completely different. The DNA-B of the East African EACMV isolates is ± 85% homologous to the West African isolates. The pairwise profile (Fig. 8B) showed the same recombinant fragment of about 800 nts with above 90% identity with West African isolates of EACMCV and other East African isolates such as EACMV-UG3, EACMZV and SACMV. The rest of the genome showed greater relatedness to the West African isolates of EACMCV, above the "species threshold" limit. Overall, the EACMCV-[TZ1] B component can be considered a non-closely related strain of the B component of EACMCV-[CM], but much closer than the B components of other East African cassava viruses.

#### iii) Comparisons of the common regions (CRs) of EACMCVs from Cameroon and Tanzania

The common region of A components (CRAs) were 82% to 89% identical to those of West African isolates, which is low but not abnormal as the West African isolates were 91% identical to one another (Table 4). The differences are mostly in the variable region between the TATA box and the TAATATTAC stem-loop, but also in the rest of the sequence. The CR of B components (CRBs) of the EACMCV-[TZ1] isolate was more distantly related, at between 78% and 80% homology to the CRBs of the West African isolates, while they were 97% homologous to one another. The differences were mostly in the variable region. When both (CRAs and CRBs) were compared, it was apparent that CRs of the East African isolates were more similar to the CRAs of West Africa than the CRBs of West Africa. This arises mainly from a deletion of GAAAA, and from a more similar sequence in the region between the TATA box and the stem-loop. The putative replication protein binding sequences (iterons) were GGTGG-AAT-GGGGG for all the isolates except for the Bs of West Africa where it is GGTGG-AAC-GGGGG. There is a repeat of GGGGG in the 5' end of the CRs for all the isolates (Fig. 6B).

### Recombination analysis of cassava mosaic geminiviruses

**Figure 9:**
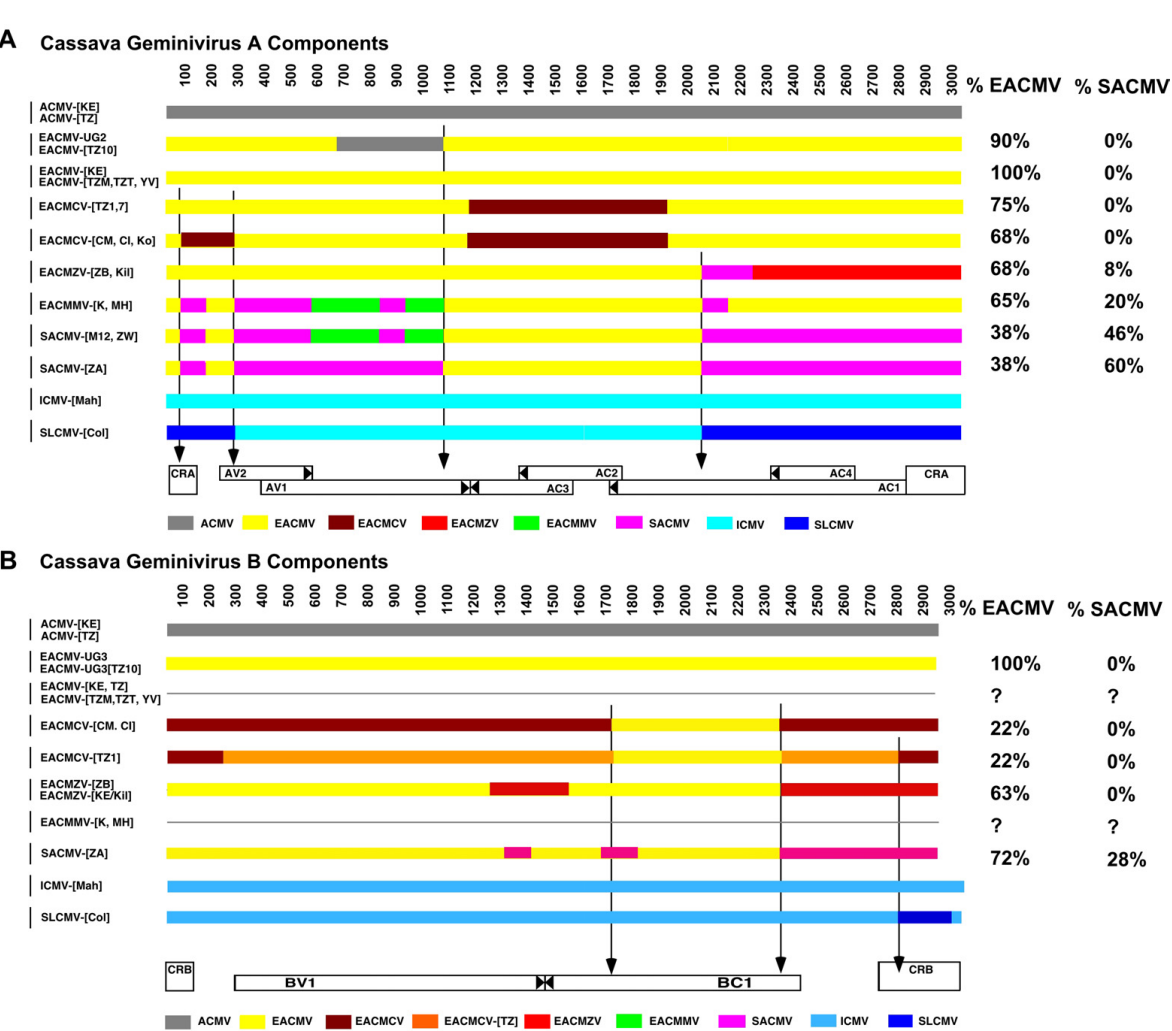
Recombination linearized map of putative recombinant fragments for the A (top) and B (bottom) components of cassava mosaic geminiviruses. Each horizontal line represents the genotype of one virus isolate and the color-coded boxes represent the tentative origins of the putative recombinant fragments. The length of the genomes is indicated on the top of each diagram and the genome organization is depicted at the bottom, while the abbreviated names of the viruses are listed on the left. The color code for the recombinant fragments is indicated in the boxes at the bottom of each diagram. The vertical arrows indicate the position of possible "hot spots" for recombination. On the right side are listed the percentages of EACMV-type and SACMV-type sequences for each virus.

The pairwise analysis performed on all African cassava viruses sequenced so far, with two Indian cassava viruses as out-groups, and including the viruses isolated in Tanzania (here described), showed a number of putative recombinant fragments for both components. Figure 9 shows a genomic map for each component and summarizes the results obtained for the A and B components.

#### i) Pairwise analysis of the A components

##### African cassava mosaic virus

None of the ACMV sequences obtained so far exhibited a possible recombinant fragment. An isolate of ACMV was involved in a recombination between EACMV and ACMV to produce the EACMV-UG2 isolate, which was associated with the epidemic in Uganda in the 90s [7,8]. But it is worth noting that ACMV acted as a donor of DNA, not a receiver, in the recombination. The situation for the EACMV-like viruses is very different, as they exhibit multiple putative recombinations between themselves and also unknown viruses. The A components of all the viruses in East Africa share a common backbone from EACMV and have integrated other pieces of DNA that have been said to originate from the other viruses not identified so far.

##### East African cassava mosaic Zanzibar virus

Two isolates of EACMZV from Zanzibar and Kenya [12] have most of their genomes from EACMV; approximately 200 nts (2050 to 2250 nts) are similar to SACMV and the rest of the genome, covering AC1, AC4 and the CR, is unique and therefore attributed to EACMZV or an ancestor of EACMZV (Fig. 9A).

##### East African cassava mosaic Cameroon virus

Several EACMCV isolates from Cameroon, Ivory Coast and now Tanzania (this report) belong to the species *East African cassava mosaic Cameroon virus *(see paragraph 3.6; [9]); all share the same putative recombinant fragment, *i.e. *a fragment of 800 nts (AC3-AC2-CterAC1), that is unique and therefore attributed to EACMCV (Fig. 9A) or a common ancestor. However, the three isolates from West Africa do have a small recombinant fragment (100–250 nts) that is also unique to EACMCV, but this fragment is not present in the Tanzanian isolates.

##### East African cassava mosaic Malawi virus

Two virus isolates from the species *East African cassava mosaic Malawi virus *from Malawi (EACMMV-[K], -[MH]) [15] show a similar recombination pattern. The first 1000 nts have either a similar pattern as SACMV-[M12] and SACMV-[ZW] or share two fragments of 100 and 750 nts with the SACMV-[ZA] isolate from South Africa (Fig. 9A). The fragments 550–800 and 900–1050 nts are therefore attributed to EACMMV or an ancestor. The major difference with the SACMV isolates resides in the fact that the rest of the genome is purely EACMV-like, with the exception of 100 nts in the AC1 gene (1950–2050 nts).

##### South African cassava mosaic virus

One virus isolate of the species *South African cassava mosaic virus *from South Africa (SACMV-[ZA]) [16] exhibited a putative recombination, *i.e. *most of the first 1000 nts (CR, AV2 and most of AV1) and then the last 800 nts (NterAC1, AC4 and CR) are unique for this virus and consequently attributed to SACMV, or an ancestor of SACMV. The rest of the genome, covering AC3-AC2 and the C-terminus of AC1, is typical of EACMV (Fig. 9A). Another two isolates of SACMV, one from Madagascar (SACMV-[M12]) and one from Zimbabwe (SACMV-[ZW]), although belonging to the same species as the virus from South Africa, have a different recombination pattern, *i.e. *the first 1050 nts are similar to EACMMV with portions that are SACMV-type and portions that are EACMMV-type (Fig. 9).

The SLCMV-[Col] and ICMV-[Mah] isolates, here used as out-groups [17], exhibited a large recombinant fragment of 1200 nts, possibly originating from ICMV [18] and encompassing NterAC1, AC4 and all the CR.

Noticeably, several recombination sites are aligned among the different genomes, possibly indicating "hot spots" for recombination and possibly also delineating fragments in which variation led to selective evolutionary advantage.

#### ii) Pairwise analysis of the B Components

The B components of CMGs also showed the presence of putative recombinant fragments as determined by the pairwise analysis. Unfortunately, some B components, such as those of EACMV-[TZ], EACMMV-[K] and -[MH], have not been cloned yet and therefore we have only partial information. The ACMV B sequences available did not show any recombination. The EACMCV isolates from Cameroon, Ivory Coast and Tanzania all showed the same putative recombinant fragment, *i.e. *between 1700 and 2300 nts, corresponding to part of the BC1 gene. Interestingly, and *a contrario *to the EACMCV A component, most of the B genome is unique and only the recombinant fragment originates from EACMV (Fig. 9B); the rest of the genome is therefore marked as the EACMCV-type (Fig. 9B). Furthermore, a comparison of the B components of the EACMCV isolates from Cameroon or Ivory Coast with the sequence from Tanzania shows between 250 and 1700 nts and between 2350 and 2800 nts, a different sequence, indicating either another two recombinations with another unknown virus or viruses, or, as supported by the number of point mutations, an extremely old sequence compared to the West African isolates of EACMCV (Fig. 9B); therefore it is marked EACMCV-[TZ]. On the contrary, the partial sequence of the B component of an isolate from Zanzibar (EACMZV-[ZB]) showed almost complete identity with a B component from EACMV-UG3, with a very short EACMZV-type fragment of 150 nts at the end of the genome. Similarly, the sole isolate of a B component of SACMV-[ZA] was almost entirely identical to EACMV-UG3, with a 500 nts fragment SACMV-type (1700 – 2300 nts), mostly corresponding to a non-coding fragment of the virus. ICMV and SLCMV B components, here used as out-groups, were essentially identical with the exception of 200 nts covering the CR of SLCMV and justifying the claim that the SLCMV A component captured the B component of ICMV [17].

### Quantification of the percentage of EACMV-type and SACMV-type sequences in each virus

**Figure 11:**
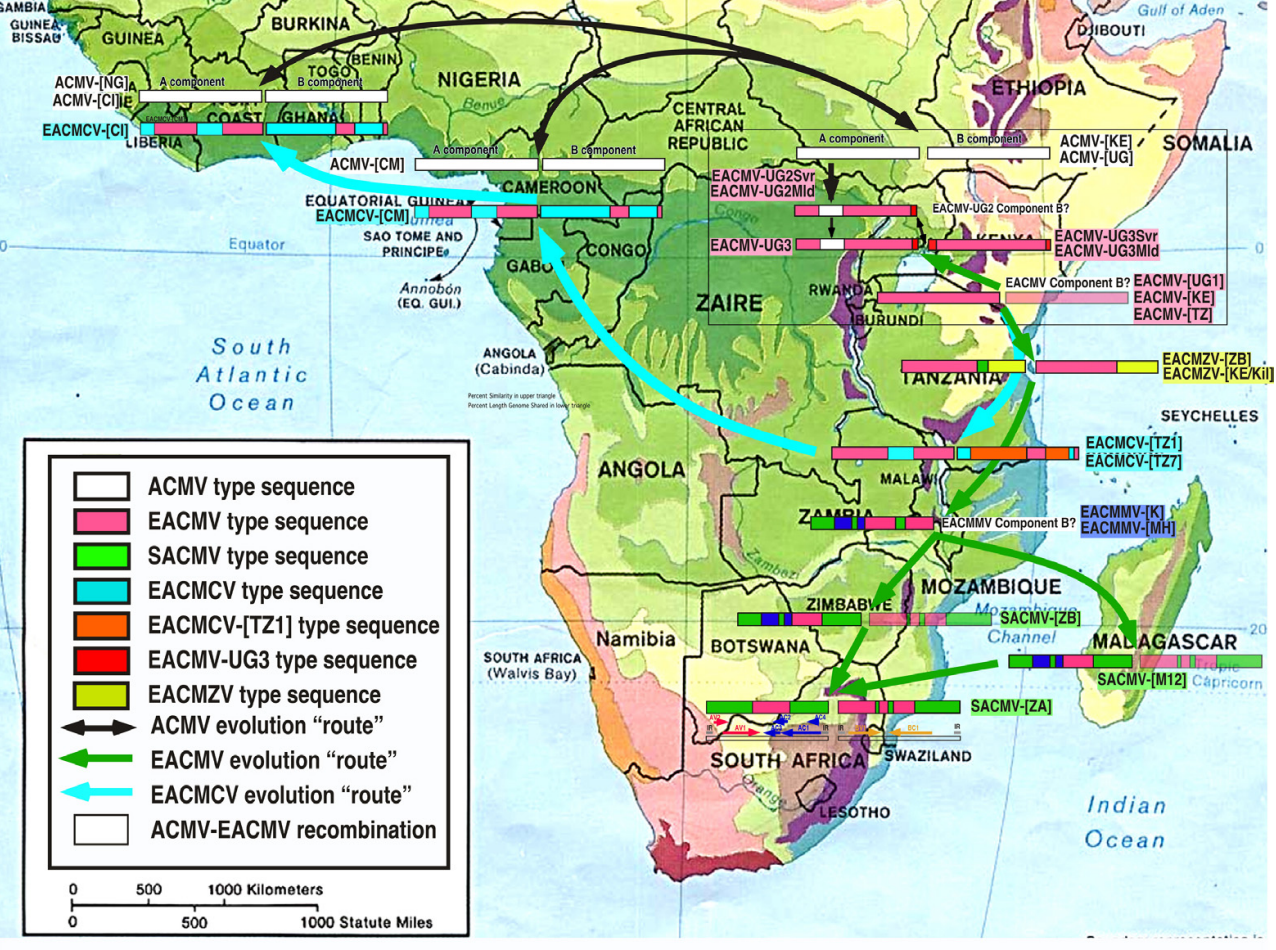
Map of Africa depicting the putative inter-species recombinations of components A and B of cassava mosaic geminivirusess identified in different parts of Africa, either from this study or from GenBank accessions. The significance of the color codes is given in the figure. Where the component B of a particular virus has not been cloned, it is indicated in letters for a different species representative or as a faded drawing for a different isolate. For simplification of the drawing, not all the ACMV isolates have been shown as they are very similar. Similarly, the EACMV-UGs associated with the CMD pandemic now present in several central African countries have not been depicted as they are of very recent introduction (less than 10 years). The solid blue arrows represent the possible "route" of evolution of the EACMCV viruses, and the green arrows represent the possible "route" of evolution of the EACMV viruses.

From the recombination analysis and phylogenetic results, it is clear that all EACMV-like viruses share a portion of the EACMV backbone sequence. The recombination map was used to calculate these percentages, indicated in Figure 9 for each component. This percentage varies from 38 to 100% depending on each virus for the A components and from 22 to 100% for the B components. A similar calculation can be made for sequences that are SACMV-type and the results vary between 0 and 60% for the A components and from 0 to 16% for the B components (Fig. 9). Figure 11 shows a repartition of these percentages according to the different viruses cloned and according to a transect between Uganda and South Africa, indicating that the EACMV backbone sequence decreases towards South Africa while the SACMV-type sequence increases.

## Discussion

The present study confirmed the presence of representatives of 3 species of CMGs in Tanzania: one isolate of ACMV, four isolates of EACMV, and two additional isolates of EACMCV. The complete DNA-A nucleotide sequences of these isolates were determined.

### ACMV

It is apparent from the results of this study that several CMGs exist in Tanzania showing a high genetic diversity. The ACMV characterized from Tanzania was found to have very high overall DNA-A nt sequence identity to all the other isolates of ACMV sequenced so far. As there is no relation between the origin of ACMV isolates and their sequence relationship with other isolates, it is impossible to tell if the one found in Tanzania is more related to one ACMV isolate than another. As it is the first isolate to be sequenced from Tanzania, we named it ACMV-[TZ]. This virus, like all the other ACMVs, displayed no detectable recombination in its DNA-A genome.

### EACMCV

**Figure 10:**
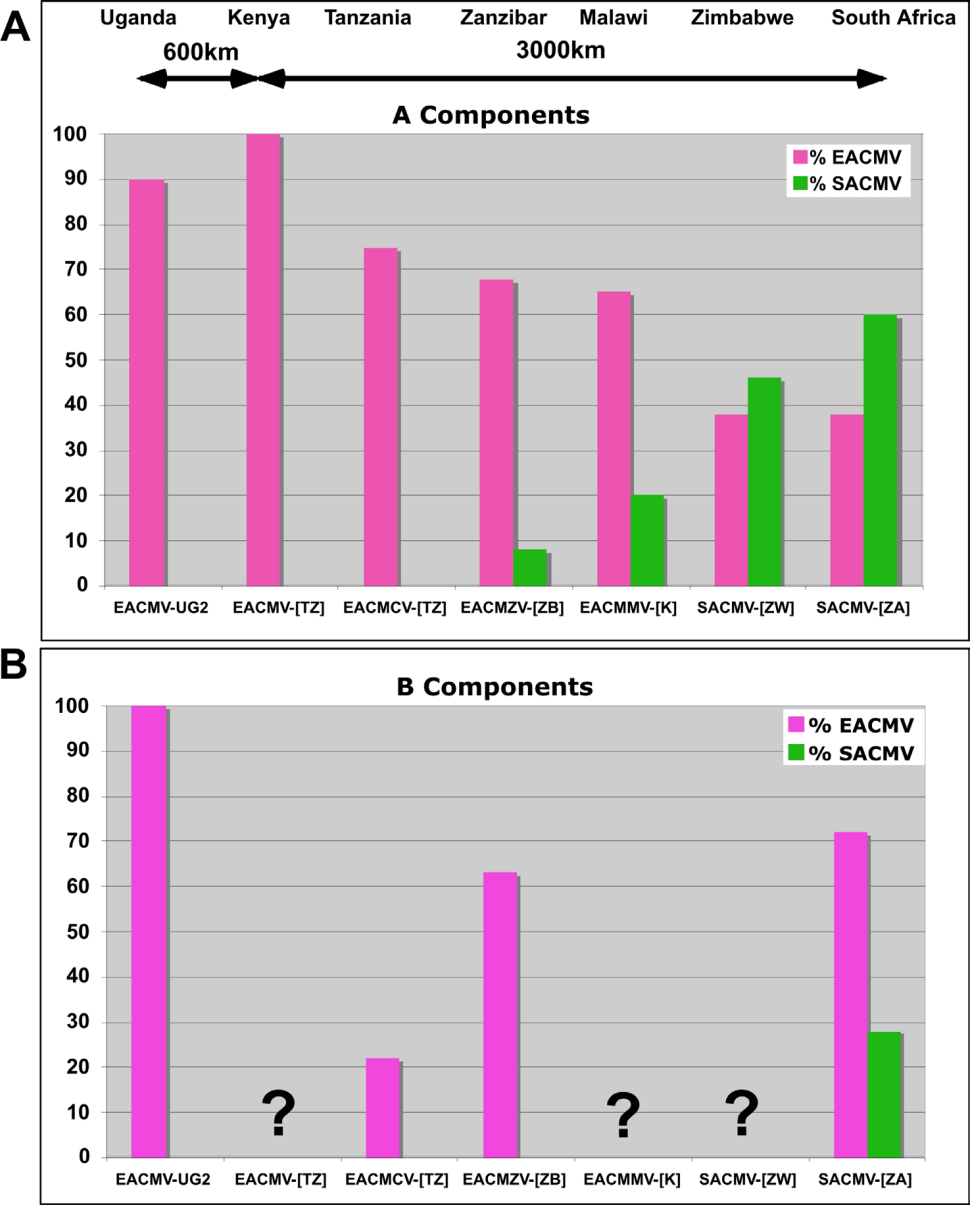
Graph representing the proportion of EACMV-type and SACMV-type sequences in each virus isolated along a transect from Uganda (Left) to South Africa (Right) for their A components (A) and B components (B). The virus name abbreviations are given in the legend of Figures 3 and 4 and throughout the text.

EACMCV-[TZ1] and EACMCV-[TZ7] had high overall DNA-A nt sequence identities, as well as high CP and CR sequence identity to members of the species EACMCV from West Africa, confirming their relatedness to that species. The two isolates from Tanzania are about 8% different, while each of them is more than 10% different to any of the West African isolates. The two Cameroonian isolates are very close to one another (>99%) and about 3–4% different from the Ivorian isolate. In addition, the Tanzanian viruses showed the same recombination, relative to EACMV-type sequences, as the EACMCVs from Cameroon and Ivory Coast, covering the C-TerAC1-AC2-AC3 region. However, we noted that the Tanzanian isolates have lost or never acquired a small recombinant sequence at the beginning of the genome, as present in the West African isolates. The EACMCV-[TZ1] B component showed the same recombination as the EACMCV-[CM] and EACMCV-[CI] B components, covering part of the BC1 region. However, the EACMCV-[TZ1] B component had an additional two putative recombinant fragments (250–1700 nts and 2350–2800 nts) not present in the West African isolates. Considering the overall sequence identity of both components, the fact that sequence differences are scattered all along their genomes and the fact that there are differences in patterns of recombination, it is strongly suggested that the two sets of viruses from East and West Africa have been separated for a very long time and are not the result of a recent introduction in either direction. One recombination in DNA-A and one in DNA-B, as they are identical, pre-date their separation, though it is not possible at this stage to date the separation. EACMCV-[TZ1] occurred widely in southern Tanzania, being present in over 98% of CMD-diseased samples collected from the southwestern part of Tanzania in the Ruvuma Region close to Lake Malawi in the same area where EACMCV-[TZ7] was found. The fact that the two sequences in Tanzania show from two to three times more sequence variability and two extra recombinant fragments, together with the fact that the parent EACMV has not been found so far in West Africa, suggests an East African origin of this virus species, and therefore a possible spread from the East to the West as indicated in Figure 10.

### EACMV-TZ, -KE, -UG

The rest of the CMGs cloned in this study were closely related to those reported in the neighboring countries of Uganda, Kenya or the previously characterized Tanzanian isolate of EACMV. These were EACMV-[TZ/YV], which resembled the EACMV-[TZ] characterized previously [19], and EACMV-[KE/TZT] that showed high sequence identity with EACMV-[KE/K2B] from Kenya, on the basis of their overall DNA-A nt sequences. While the CP of EACMV-[TZ/YV] showed high sequence identity with EACMV-[TZ] and EACMZV-[ZB] from the island of Zanzibar [12], EACMV-[KE/TZT] from Tanga region showed high nt sequence identity with its close relative EACMV-[KE/K2B]. Similarly, EACMV-[KE/TZM] also shared high CP nt sequence identity with EACMZV-[ZB]. It was found in only ten samples and very localized in spread within the region. Plants singly-infected with EACMV-[KE/TZM] expressed very severe symptoms both in the field and growth chamber. Whether this phenotype was a result of the nature of the EACMV-[KE/TZM] DNA-A genome remains to be established. The EACMV-UG2 [TZ10] shared very high DNA-A and CR sequence identity with EACMV-UG2Svr from Uganda. The CR also showed 100% nt sequence identity with EACMV-UG2Svr as well as high CP sequence identity to ACMV isolates because it has the same recombination as its closest relative, EACMV-UG2Svr, that was proven to involve two viruses (ACMV and EACMV) [7,8]. This CMG was localized in the northwestern part of Tanzania in the post-epidemic area. It is noticeable that this virus, which has invaded a large portion of Central Africa in just a few years [4] (Fig. 8), has not yet reached the southern and eastern part of Tanzania.

### Recombination of A and B components

Using all the CMG sequences available so far, we have shown that both A and B components of most of the CMGs exhibit putative recombinant fragments from various known or unknown origins. Despite the smaller number of sequences of DNA-B components and the smaller number of putative recombinant fragments, it is interesting to note that, as for the A components, it seems that there are "hot spots" for recombination. These apparent hotspots for recombination could result from physical constraints in the virus sequences or could simply result from the functional constraints of having recombinant proteins that keep structural and biological functions. These hotspots have already been mentioned in other general studies of geminivirus recombination [14] as well as in specific studies of particular groups of geminiviruses [20].

### Two categories of CMGs in Africa

Based on recombination analyses, it is apparent that there are really two different categories of CMGs. The ACMV group does not have fragments of foreign geminivirus DNA in their genomes. By contrast, all other African CMG species groups show evidence of extensive recombination. It is also significant that EACMCV isolates obtained from each side of the African continent appear to share a similar genetic make-up and recombination pattern. This suggests that these viruses had a common origin, probably in East Africa, but diverged a long time ago. Recombination events have been shown to be key factors in the development of CMD epidemics [7,8,19] and it has been suggested that recombination is a significant contributor to geminivirus evolution [14]. Recombination involving the CP sequence has been reported for EACMV-UG2 from Uganda [7,8,10], a virus that has been associated with the current CMD pandemic that has devastated cassava in eastern and central African countries [4,21], although there is currently no proof that this event has been the key factor driving the pandemic's spread.

### B components of CMGs in Tanzania

The diversity of DNA-B components of EACMV from Tanzania was investigated using partial DNA-B nt sequences (BC1-CR) of ~560 bp. Generally, there was little genetic divergence among the compared isolates with the exception of TZB6 that shared 97% sequence identity to EACMV-UG1 (AF230375) and 96% with EACMV-UG3 from Uganda. Isolate TZB1 and TZB7 clustered with EACMCV-TZ1 and are probably Bs of EACMCV A components. However, for the other isolates that grouped or formed their own group in the phylogenetic analysis, it was difficult to speculate as to what they represent partly because the DNA-Bs of EACMV-[TZ], EACMV-[KE] and EACMMV have yet to be sequenced. However, these short fragments indicated a clustering, apart from EACMV-UG and EACMCV, into 4 additional clusters that could reflect an even greater molecular diversity in the B components of CMGs in East Africa than we currently recognize.

### EACMV evolution

The clustering of all the EACMV-like viruses into one species has been the topic of much scientific debate in recent years. ICTV (International Committee on Taxonomy of Viruses) finally decided to split them into 5 species (for now), mostly to comply with the ICTV guidelines for species demarcation, but clearly these viruses are closely related and had common ancestors. All EACMV-like viruses with the exception of EACMCV occur in East Africa, and mostly east of the Rift Valley. Evidence presented here and elsewhere now provides a strong case for an East African origin for the EACMVs. EACMCV is widely-distributed across West Africa, albeit at low incidence [4]. Whilst it seems likely that this is the result of an early introduction or introductions from East Africa, it is not currently clear when such an introduction(s) might have taken place. It is even possible that the spread of this virus occurred in another host, long before cassava was introduced into Africa.

Finally, the rapidly expanding EACMV-UG2 associated pandemic of severe CMD in East and Central Africa represents a contrasting, and currently probably unique, scenario in which the combination of a virulent recombinant virus, superabundant vector populations and susceptible local cassava germplasm have led to a rapid expansion in the geographic range of EACMV-UG with a concomitant devastating impact on cassava cultivation. Furthermore, it is significant that when considering the proportion of pure EACMV backbone sequences in the A components of all the EACMV-like viruses, there is a clear gradient from East Africa to South Africa, going from 100 to 38%, suggesting firstly that these viruses are highly related and secondly that the origin of the EACMVs might have been East Africa, hence the green arrows in Figures 8 and 11. Similarly, a reverse gradient for the SACMV-like sequence, going from 8 to 60% from Zanzibar to South Africa, suggests that the SACMV ancestor was located in South Africa. Because recombination can only occur when the two parent viruses are in the same plant, it is logical to expect a spatial relation between the different viruses and their genetic make up. It is, however, the first time that such gradients have been demonstrated for geminiviruses. The situation for the B components is completely different. There is no EACMV-like gradient from North to South, as most of the available sequences show a great proportion of EACMV-like sequences. However, it is evident that EACMCV has captured a B component completely different from EACMV, with only a small EACMV-like fragment. This result is concordant with the idea that B components can be recruited independently from the genetic nature of A components as already suggested for SLCMV and ICMV [17].

### East Africa

East Africa has been the cradle for many biological organisms beginning with humanity. From this work, it is also apparent that Tanzania may also be a potential source of origin of the family of EACMVs. The revealed strain diversity further exemplifies the wealth of this part of Africa with respect to cassava geminiviruses. Some of these viruses have been introduced very recently, such as EACMV-UG2 [TZ10], while others, such as ACMV and the EACMVs, have clearly been present much longer. The East-African Arc Mountain is known to be the main bio-diversity hot-spot in Africa, and an important refuge for plants and animals [22], therefore it is plausible that some of the geminiviruses that were invading local host plants were spread throughout Africa in their local hosts (for many millions of years), as it was suggested for *Rice yellow mottle virus *[23] colonizing the domesticated host in very recent history (a few hundred years). The same type of geminiviruses would have colonized cassava wherever they might be, beginning with that crop's introduction into the African continent in the XVI^th ^century, as these viruses would have had the same potential for such colonization. This might have been the case for EACMCV for which our data presented here suggest an old East African origin for the now widely distributed EACMCV in West Africa. In addition to this scenario, it is certain that cassava geminiviruses have been exchanged throughout the movement of virus infected cassava cuttings via human intervention and by the natural vector *Bemisia tabaci*. The latter may account for the EACMV/SACMV gradient between East Africa and South Africa, favoured by a natural corridor along the eastern Rift Valley and created by the recombination capacity of CMGs present in the same region.

However, more sequences are required in order to compare and contrast variability within and between the virus populations and to strengthen the understanding of their evolutionary interrelationships. The rapid spread of the EACMV-UG2 associated pandemic has been driven through superabundant whitefly populations [24], but other important forces in CMG movement and evolution include movement of cassava cuttings and transmission from and into alternative weed hosts. Although cassava was brought to Africa in the XVI^th ^century, it attained its current Africa-wide distribution as recently as the XIX^th ^century. Most current movement occurs informally as farmers move cuttings locally. Wider distribution is less frequent but may be more significant in enabling major displacements of CMGs, such as that hypothesized for the introduction of EACMCV from East to West Africa. Although rapid spread of up to 100 km per year has been reported for the EACMV-UG associated pandemic [4], elsewhere there appears to be much less local spread of CMGs by whitefly, and physical barriers including lakes, forests and regions where cassava is not grown, appear to be effective in curtailing local spread of CMG. This would seem to account for the apparent 'island' of EACMCV in southern Tanzania as well as the absence of ACMV from coastal East Africa.

## Conclusion

In conclusion, we have established the existence of different CMG isolates, strains and species in Tanzania with some isolates resembling those reported previously in East African countries and two isolates very similar to the geographically distant EACMCV from West Africa. This study demonstrates that East Africa is rich in CMGs and could be the cradle for CMG diversification in Africa. It also highlights the urgent need for more information. Only through building a thorough understanding of these important plant pathogens and the evolutionary processes underpinning their emergence can we hope to develop effective and sustainable approaches to managing the disease they cause.

## Methods

### Collection of plant samples

A total of 510 samples were collected during September 2002 from the northeastern coast (60), east coast (74), southeastern coast (68), southern region (70) and the Lake Victoria Basin (238), representing the major cassava-growing areas in Tanzania. Cassava leaf samples and cuttings (25–30 cm in length) were collected from plants expressing CMD symptoms in fields located at a minimum of 5 km intervals. Leaf samples were kept in a cool box for DNA processing. Selected cassava cuttings were transported to the Donald Danforth Plant Science Center, St. Louis, MO for replanting in controlled growth chambers.

### Symptom reproduction in the growth chamber

Selected cassava cuttings collected from the fields were planted in a growth chamber at 25°C with a 16 hours day length and 50% relative humidity and watered twice weekly. CMD symptoms were recorded daily on the newly formed leaves for the first three months and every three days in the subsequent months for an eight month period. Symptom severity on the top five fully-expanded leaves was scored using a scale described by Fauquet *et al *[25].

### DNA extraction

Total DNA was extracted from the symptomatic cassava leaves collected in the field and growth chamber as described by [26].

### Polymerase chain reaction, cloning, and sequencing

Full-length copies of DNA-A were amplified from total cassava plant DNA extracts using sets of primers (Table 1). UNI/F and UNI/R are degenerate primers with annealing positions in the AC1 gene designed to amplify near full-length DNA-A of CMGs (2.7–2.8 kbp) leaving an unamplified portion of ~17 nts. From the near full-length CMG sequences, primers were designed to amplify the remaining partial DNA-A sequences including the missing 17 nts from the original samples. Partial fragments consisting of a region between the BC1 gene and intergenic region (IR) of DNA-B components of EACMV isolates from different cassava-growing areas were amplified by universal primers EAB555-F and EAB555-R (Table 1) designed to amplify PCR products of about 540–560 kbp depending on the virus isolate. In order to amplify the DNA-A and DNA-B full-length, PCR was performed with 94°C denaturation followed by 35 cycles of 1 min at 94°C, 59°C for 1 min and 2 min at 72°C. For amplification of the partial DNA-B fragment (BC1/IR), PCR conditions were 30 cycles of 94°C for 1 min, 55°C for 1 min, 72°C for 1 min and an extension cycle of 10 min at 72°C. PCR products of the expected sizes were electrophorezed in a 1% agarose gel in TAE buffer (10 mM tris-acetate, 1mM NaEDTA, pH 8.0), purified, and cloned into the pCR 2.1 vector using the TA cloning kit (Invitrogen, San Diego, CA). Clones containing putative viral sequences were identified by miniprep screening and confirmed positive for inserts by PCR amplification using their respective PCR primers, and inserts were subsequently sequenced in both directions. The complete and partial nucleotide sequences of CMGs were determined by the dideoxynucleotide chain termination method using an ABI automatic sequencer on both orientations at the Protein and Nucleic Acid Chemistry Laboratories (PNACL), Washington University School of Medicine, St. Louis, Missouri, USA (ABI377 DNA sequencer, Perkin Elmer, Foster City, CA). Sequence fragments of < 600 kbp were generated using M13 universal primers. Moreover, to obtain overlapping data from opposite strands of large or full-length fragments, single primers were constructed for genome walking. Sequences were submitted to GenBank and the accession numbers are as follows: Complete nucleotide sequence of DNA-A named EACMCV-[TZ1] (AY795983); EACMCV-[TZ7], (AY795984); EACMV-UG2 [TZ10], (AY795988); EACMV-[KE/TZM] (AY795986); EACMV-[KE/TZT], (AY795985); EACMV-[TZ/YV], (AY795987); ACMV-[TZ] (AY795982); and DNA-B for EACMCV-[TZ1](AY795989). Partial DNA-B (BC1/ICR) sequences of EACMV isolates from Tanzania named TZB (AY800251), TZB1 (AY800252), TZB2 (AY800253), TZB3 (AY800254), TZB4 (AY800255), TZB5 (AY800256), TZB6 (AY800257), TZB7 (AY800258), TZB8 (AY800259), TZB9 (AY800260), TZB10 (AY800262), TZB11 (AY800261), and TZB12 (AY800263).

### Computer analysis of CMG sequences

Virus sequences were edited using BioEdit Sequence Alignment Editor (Hall, 1999) and SeqEdit (DNAStar, Madison, WI) to obtain a consensus sequence for each. Reference geminiviruses for full length CP and CR sequence alignments were compiled by extracting the complete DNA-A and DNA-B sequences, the CP ORF (approximately 765–777 bp) and CR sequences (approximately 150–170 bp) from sequences available in GenBank (accession # are provided in the figures). Multiple sequence alignments of the full-length DNA-A, DNA-B, capsid protein (CP) gene and common region (CR) were carried out using the Clustal Program (MegAlign, DNAStar). The phylogenetic trees were constructed from the multiple alignments by the neighbor-joining majority rule consensus. Multiple alignments were analyzed by maximum parsimony with full-length DNA-A, DNA-B and CP phylogenetic trees using Phylogenetic Analysis Using Parsimony (PAUP) [27] and a bootstrap analysis with 1000 replicates was performed. Only values above 50% were reported on the trees in the figures. Virus specific iterons in the CR of selected CMGs were identified and compared with the analogous iterons of the Tanzanian isolates of CMGs.

### Recombination analysis for cassava mosaic geminiviruses

**Figure 1:**
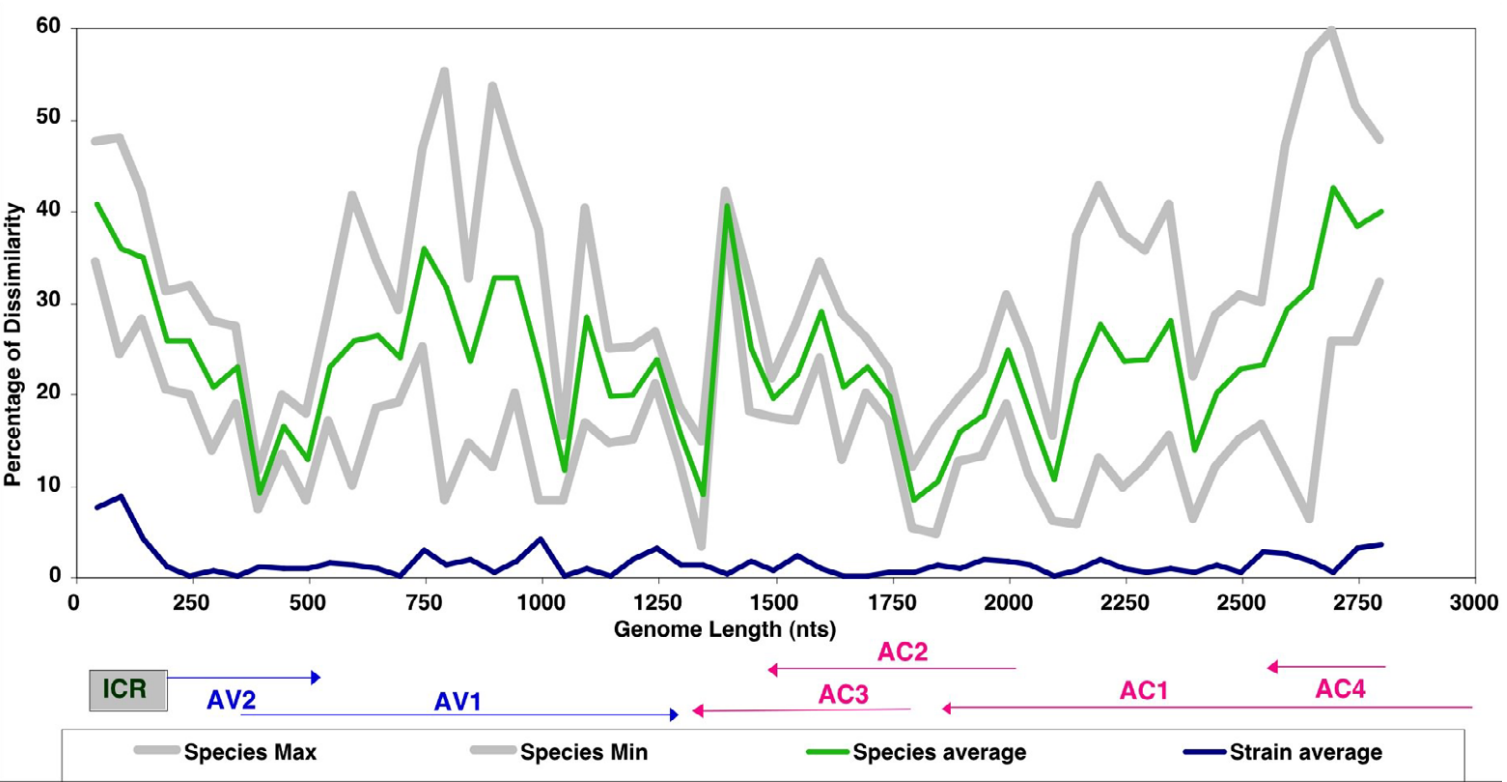
(A) Pairwise analysis of begomoviruses in the Old World that do not exhibit putative recombinant fragments at the species level (green curve) and at the strain level (blue curve). (B) Pairwise analysis of EACMCV-[TZ1] A component, paired with the sequence of the A component of other cassava mosaic geminiviruses like EACMCV-[TZ7] (blue line), ACMV-[TZ] (brown line), EACMV-[KE] (red line) and EACMZV-[ZB] (green line), showing the recombinant fragment of this virus (1200 – 2000 nts) as well as the one from EACMZV-[ZB] (2000 – 2900 nts). The linearized genome organization of these geminiviruses is depicted at the bottom of the graph.

The pairwise comparison sequence analysis (PCSA) method compares the profile of a pair of sequences to that of an average profile of sequences that are selected *a priori*, based on knowledge that the selected sequences are related to the species or the isolate levels [20]. According to the guidelines established by the ICTV *Geminiviridae *Study-Group, two geminivirus sequences sharing more than 89% identity of their A component sequences are considered strains or isolates of the same species. Where homology is less than this, they are considered to be members of different species [5]. However, viruses that share between 80 and 90% sequence identity are often found to be recombinants [2], therefore, in the PCSA, we consider viruses sharing less than 80% identity as different species. PCSA profiles were carried out between sequences of different species and of different isolates and an average profile for the considered cluster of viruses was calculated for these two categories with increments of 50 nts along the genome sequence. A standard deviation value for each segment was calculated and minimum and maximum values corresponding to two standard deviation values were also calculated (Fig. 1). Each chosen pairwise analysis for putative recombinant sequences was then compared to the species average profile and the pertaining of each 50 nts fragment to this category is examined. Segments different from more than 2 standard deviation values were considered to be putative recombined fragments. For each PCSA, a putative recombination percentage for the genome is calculated and a corresponding map can be drawn. It is verified (*a posteriori*) that the particular representatives of species and isolates selected for the 'Species and Isolate Average Curves' are 100% non-recombinant at the time of the analysis [20]. No statistical test is applied to PCSA.

## Competing interests

The author(s) declare that they have no competing interests.

## Authors' contributions

Design and conception of the study (JN, JPL, TASA, GT, CMF); execution of the experiments (JN); manuscript preparation (JN, JPL, CMF); sequence analysis, alignment and phylogeny (JN, CMF). All authors read and approved the final manuscript.
